# Supplementary material for: Causal interplay between lactose intolerance and gut microbiota: a combined bidirectional Mendelian randomization and in vivo validation study
Source: Front Nutr. 2026 Jun 1;13:1803337. doi: 10.3389/fnut.2026.1803337 (PMC13265576; doi:10.3389/fnut.2026.1803337)
Supplement: Supplementary file 1 [file Data_Sheet_1.zip › supplementary materials/Forward/forest plot/ebi-a-GCST90027710.finngen_R12_E4_LACTONAS.pdf]

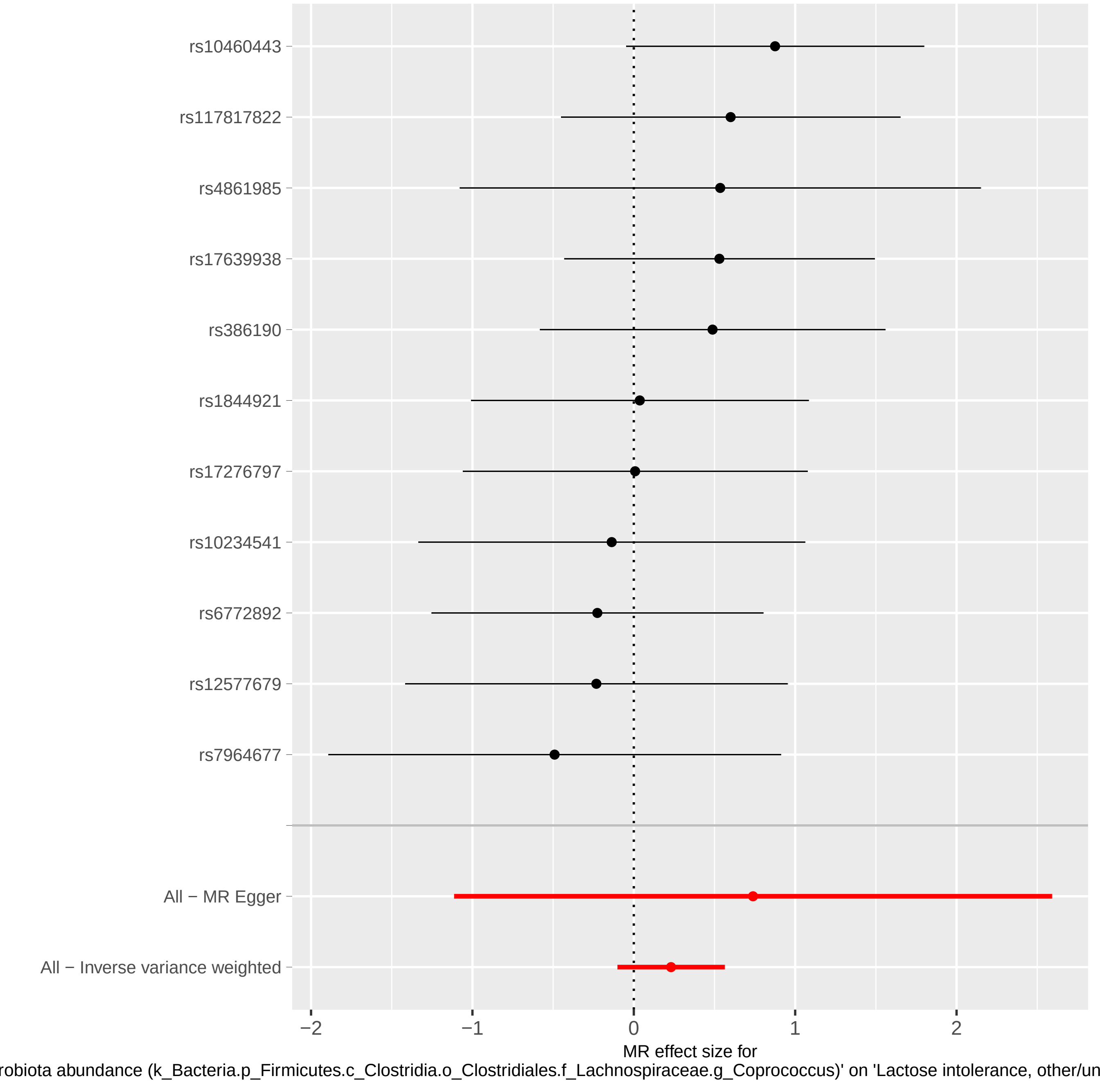

rs10460443
rs117817822
rs4861985
rs17639938
rs386190
rs1844921
rs17276797
rs10234541
rs6772892
rs12577679
rs7964677
All − MR Egger
All − Inverse variance weighted
−2
−1
0
1
2
MR effect size for
robiota abundance (k_Bacteria.p_Firmicutes.c_Clostridia.o_Clostridiales.f_Lachnospiraceae.g_Coprococcus)' on 'Lactose intolerance, other/un
